# The Diagnostic Performance of Minimally Invasive Biopsy in Predicting Breast Pathological Complete Response After Neoadjuvant Systemic Therapy in Breast Cancer: A Meta-Analysis

**DOI:** 10.3389/fonc.2020.00933

**Published:** 2020-06-26

**Authors:** Yan Li, Yidong Zhou, Feng Mao, Yan Lin, Xiaohui Zhang, Songjie Shen, Qiang Sun

**Affiliations:** Department of Breast Surgery, Peking Union Medical College Hospital, Peking Union Medical College, Chinese Academy of Medical Sciences, Beijing, China

**Keywords:** breast cancer, neoadjuvant, minimal invasive biopsy, pathologic complete response, meta-analysis

## Abstract

**Background:** Neoadjuvant systemic therapy (NST) is commonly used in patients with early stage breast cancer before definitive surgery. The standard diagnostic approach for pathologic complete response (pCR) of the breast is breast surgery and pathologic examination. In recent years, several trials investigated the predictive value of image-guided minimally invasive biopsy (MIB) for breast pCR after NST. This study conducted a meta-analysis to evaluate the diagnostic accuracy of MIB.

**Materials and Methods:** We identified relevant research reports in online databases through February 2020. The Quality Assessment of Diagnostic Accuracy Studies 2 (QUADAS-2) tool was used to evaluate the quality of included trials. We extracted relevant data and constructed a 2 × 2 contingency table to analyze the predictive accuracy of MIB for breast pCR. Subgroup analyses and meta-regressions were also performed to investigate potential causes of heterogeneity.

**Results:** Nine trials (with 1,030 breast cancer patients) were included in this meta-analysis. The pooled sensitivity and specificity of MIB were 0.72 [95% confidence interval (CI): 0.61–0.81] and 0.99 (95% CI: 0.89–1.00), respectively. By combining relevant data, there were no significant differences in sensitivity or specificity among different molecular subtypes of breast cancer (*P* > 0.05). Subgroup analyses and meta-regressions implied that trials with responses not limited to clinical complete response (cCR) had a significantly higher accuracy of MIB than those with only cCR (RDOR: 7.65; 95% CI: 1.05–55.46; *P* = 0.046).

**Conclusion:** Current image-guided MIB methods are not accurate enough in terms of predicting breast pCR after NST. It is of utmost clinical importance to standardize the MIB procedure and incorporate other factors into the evaluation in order to improve the accuracy to an acceptable level.

## Introduction

Neoadjuvant systemic therapy (NST) is used in approximately 30% of patients with early stage breast cancer before definitive surgery (1). Pathologic complete response (pCR) is an ideal response to NST, indicating the absence of residual cancer in a surgical specimen although it has different definitions (2, 3). In recent years, with the improvement of neoadjuvant chemotherapy and targeted therapy, the pCR rates of breast cancer have increased dramatically. For triple-negative (TN) and human epidermal growth factor receptor 2-positive (HER2+) subtypes, pCR rates of up to 60–70% can be achieved with the administration of carboplatin regimens and dual HER2 blockage (4, 5).

Achievement of pCR after NST is associated with less recurrence and favorable survival of breast cancer, and recent studies show that escalation of adjuvant systemic therapy could have additional benefits for patients with residual disease (non-pCR) (2, 3, 6, 7). Currently, the standard diagnostic approach for pCR of the breast is breast surgery and pathologic examination of the specimen. As one of the main options of breast surgery, breast-conserving surgery after NST is universally performed, the oncologic safety of which has been confirmed by a series of studies (8, 9). For patients with no residual cancer in the breast (breast pCR), it is reasonable to consider an omission of breast surgery (10, 11). From this point of view, it is of great clinical significance to explore a less invasive method to predict breast pCR after NST.

Some studies focus on the accuracy and reliability of non-invasive imaging methods to predict breast pCR, but the results are far from satisfactory. Neither ultrasound nor mammography is reliable with false negative rates (FNR) ranging from 9 to 70% (12–14). Similarly, magnetic resonance imaging (MRI) demonstrated an FNR up to 30–50% in predicting breast residual tumor after NST (15–17). Imaging alone is not accurate enough to replace the pathologic examination of a surgical specimen.

In recent years, several trials have investigated the predictive value of image-guided minimally invasive biopsy (MIB) for breast pCR after NST, and these approaches include core needle biopsy (CNB), vacuum-assisted biopsy (VAB), and fine-needle aspiration (FNA) (18–27). With different biopsy procedures, these trials demonstrate the diverse accuracy of MIB in identifying breast pCR.

In the present study, meta-analysis is performed to assess the diagnostic accuracy of MIB in predicting breast pCR after NST. We also perform subgroup analyses and meta-regressions to find which factors are associated with the predictive capability.

## Materials and Methods

### Search Strategy and Study Inclusion

This review was conducted according to the guidelines stipulated in Preferred Reporting Items for Systematic Reviews and Meta-Analyses (PRISMA) for diagnostic test accuracy (28). We searched electronic databases, including PubMed, EMBASE, and the Cochrane library, and the latest search was performed on February 25, 2020. In addition, we searched conference presentations and abstracts, such as San Antonio Breast Cancer Symposium (SABCS), American Society of Clinical Oncology (ASCO) meetings, and the European Society for Medical Oncology (ESMO) meetings held within the last 15 years. To identify relevant studies, the following terms were employed as queries: “breast cancer,” “neoadjuvant,” “biopsy,” and “pathologic complete response (OR pCR)” as well as MeSH terms “breast neoplasms” and “neoadjuvant therapy.” We included clinical trials using the following criteria: (1) The investigation involved patients with early stage invasive breast cancer who received NST; (2) after completion of NST, the patients received MIB (e.g., CNB, VAB, and FNA) of the breast, followed by standard breast surgery (lumpectomy or mastectomy); (3) the trials report histopathologic results of both index tests (MIB) and reference standard tests (surgery) and provide measures of test accuracy (e.g., sensitivity, specificity, and false negative rate), which allowed construction of a 2 × 2 contingency table with absolute numbers of true positive (TP), true negative (TN), false positive (FP), and false negative (FN) results. Studies meeting the following criteria were excluded: (1) reviews or case reports, (2) studies focusing on non-invasive tests (e.g., imaging examinations), (3) studies focusing on axillary evaluation rather than breast, and (4) studies without sufficient data even after attempting to contact the corresponding authors.

### Data Extraction and Quality Evaluation

Data extraction and the evaluation of the quality of the studies were independently performed by two reviewers (YL and YZ). In cases of discrepancies, consensus was reached by them. The following data were extracted: first author, country of origin, update year, study design, sample size, patient characteristics, procedures of MIB, breast pCR rate, test accuracy measures, and complications of biopsy. For investigations with more than one report, data were gathered from the most recent findings.

Residual tumor was defined as “positive,” and the absence of residual tumor was defined as “negative” in both surgical and MIB specimens. For purposes of data synthesis, we extracted raw cell numbers for TP, TN, FP, and FN. If the numbers were not available, we employed the Revman calculator to generate these data based on the detailed information of test accuracy measures.

Eligible studies were evaluated for quality using the Quality Assessment of Diagnostic Accuracy Studies 2 (QUADAS-2) tool.

### Statistical Analysis

Inter-study heterogeneity was evaluated using the Cochran's Q statistics and *I*^2^*-*test. Heterogeneity was considered significant as either *P* < 0.05 or *I*^2^ > 50%. In the absence of statistically significant heterogeneity, we calculated the pooled effect using a fixed-effects model; however, with significant heterogeneity, we employed the Mantel–Haenszel random effects model.

The measures of interest for pooled analyses included sensitivity, specificity, false negative rate (FNR = 1-sensitivity), negative likelihood ratio (NLR), positive likelihood ratio (PLR), and diagnostic odds ratio (DOR). Sensitivity and specificity of each study were used to plot the summary receiver operator characteristic (SROC) curve with the area under the curve (AUC) to indicate test accuracy.

For those studies reporting detailed information of specific molecular subtypes of breast cancer, we performed pooled analyses according to different molecular subtypes (TNBC, HER2+, and HR+HER2−). We also conducted subgroup analyses and meta-regressions according to region, study design, tumor response to NST, and sample size in evaluating potential causes of heterogeneity. The relative DOR (RDOR) was used to assess the heterogeneity of subgroups.

The analysis was performed using Stata 12.0 software (Stata Corporation, College Station, TX, USA), RevMan 5.3 software (the Cochrane Information Management System), and Meta-Disc (XI Cochrane Colloquium; Barcelona, Spain). *P* < 0.05 (two-sided) was considered to be statistically significant.

## Results

### Characteristics of the Studies

**Figure 1:**
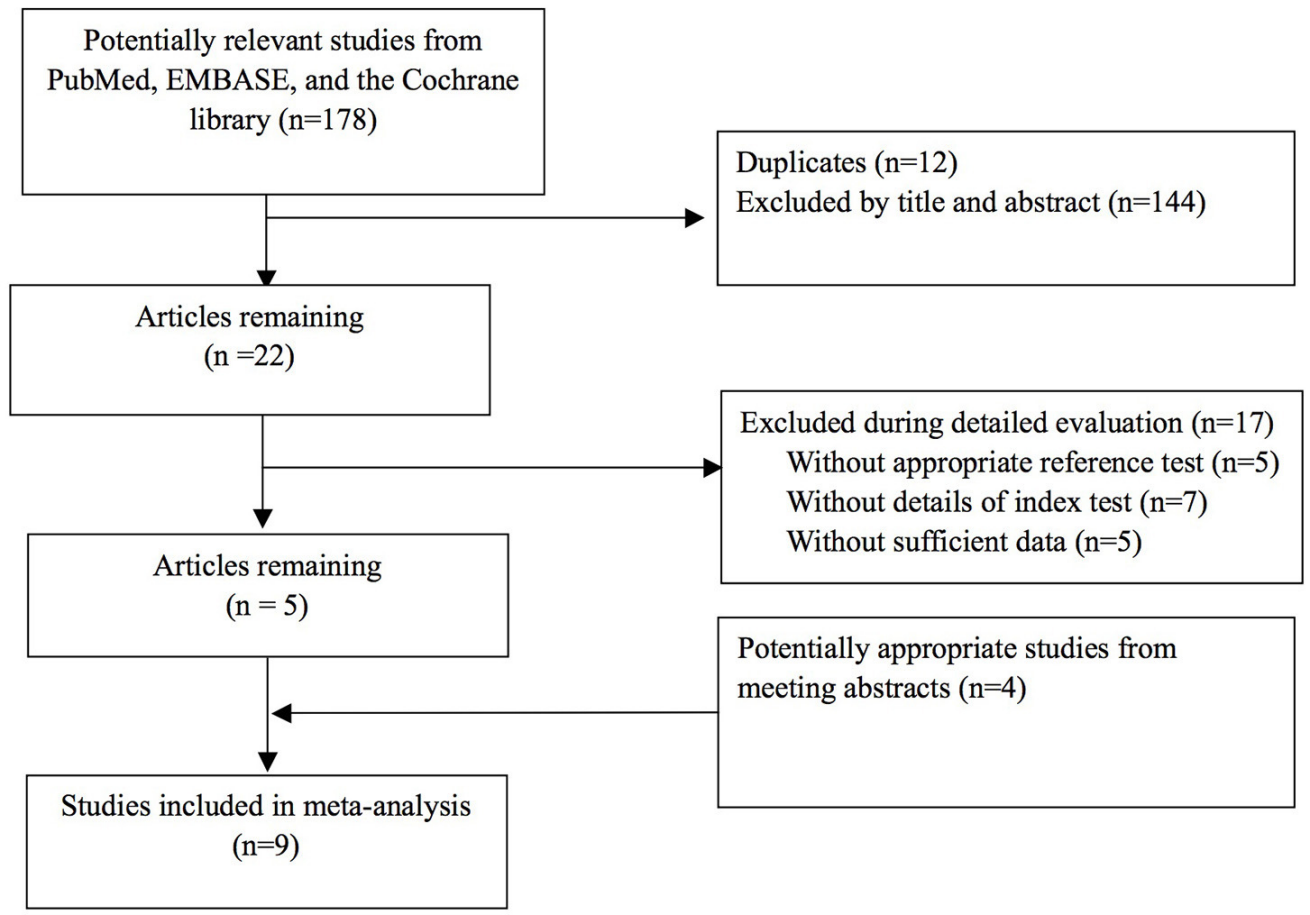
Flow diagram of the systematic search and selection process of studies.

A total of nine clinical trials fulfilled the eligibility criteria. Figure 1 shows the search and selection process used in this study. Altogether, the nine trials described a total of 1,030 patients. Among the trials included, five had published articles with full text, and the other four were reported only as abstracts at annual conferences; five were single-center studies, and four were multicenter studies; eight were prospective, and only one was retrospective; six of them were carried out in Europe, two in America, and one in Asia (Table 1).

**Table 1:** Study characteristics.

**Author/study name**	**Country**	**Update year**	**Study type**	**Number of participants**	**Response to NST before biopsy**	**MIB characteristics**	**Number of cores**	**pCR rate**	**Overall performance results**	**Results of reported subgroups**	**Complications of biopsy**
Lee et al. (27)	South Korea	2018	Prospective, single-center	40	cCR (by MRI)	Image-guided CNB (14G) or VAB (10G)	NR	67.5% (27/40)	Sensitivity = 69.2%; FNR = 30.8%; NPV = 87.1%	With ≥5 biopsy samples: Sensitivity = 88.9%; FNR = 11.1%; NPV = 96.2%	NR
Tasoulis et al. (26)	UK	2018	Retrospective, single-center	53	cPR or cCR (by imaging)	Image-guided VAB	NR	41.5% (22/53)	Sensitivity = 80.6%; FNR = 19.4%; NPV = 76.9%	TNBC: Sensitivity = 100%; FNR = 0%; NPV = 100%	NR
Basik et al. /NRG-BR005 (19)	USA	2019	Prospective, multi-center	98	cCR and rCR/near rCR (by tri-modality imaging)	Marker clip directed, stereotactic CNB	1–13	63.3% (62/98)	Sensitivity = 50.0%; FNR = 50.0%; NPV = 77.5%	TNBC: FNR = 63.6%; NPV = 74.1% HER2+: FNR = 40%; NPV = 89.5% HR+HER2−: FNR = 46.7%; NPV = 46.2%	7/98 (7.1%):6 post-procedure hematomas, 1 breast pain)
Francis et al. /NOSTRA PRELIM (21)	UK	2017	Prospective, single-center	20	No criteria of response to NST	Marker clip directed, ultrasound-guided CNB	2–6 (mean: 4)	10.0% (2/20)	Sensitivity = 77.8%; FNR = 22.2%; NPV = 33.3%	NR	NR
Kuerer et al. (23)	USA	2018	Prospective, single-center	40 (included only TNBC and HER2-amplified cases)	cPR or cCR (by imaging)	Ultrasound-guided or mammography-guided combined FNA and VAB (9G)	4–14 (mean:12)	47.5% (19/40)	Sensitivity = 95.0%; FNR = 5.0%; NPV = 95.0%	NR	8/40 (20.0%):3 post-procedure hematomas, 4 bleeding, 1 bruising)
Peeters et al. and van der Noordaa et al. /MICRA (20, 25)	Netherlands	2019	Prospective, multi-center	167	cPR or cCR (by MRI)	Ultrasound-guided CNB (8–14G)	NR	53.3% (89/167)	Sensitivity = 62.8%; FNR = 37.2%; NPV = 75.4%	cCR after NAC: FNR = 47.3%; NPV = 75.5%. cPR after NAC: FNR = 13.0%; NPV = 75.0%	NR
Heil et al. (24)	Germany	2015	Prospective, multi-center	164	cCR	Ultrasound-guided or mammography-guided CNB (14G) or VAB (9–11G)	NR	56.7% (93/164)	Sensitivity = 50.7%; FNR = 49.3%; NPV = 71.3%	TNBC: FNR = 64.7%; NPV = 75.6% HER2+: FNR = 50%; NPV = 83.7% HR+HER2-: FNR = 42.1%; NPV = 42.9%	NR
Heil et al. (22)	Germany	2016	Prospective, single-center	50	cPR or cCR (by imaging)	Ultrasound-guided VAB (9G)	6–12	46.0% (23/50)	Sensitivity = 74.1%; FNR = 25.9%; NPV = 76.7%	TNBC: FNR = 28.6%; NPV = 80.0% HER2+: FNR = 16.7%; NPV = 87.5% HR+HER2–: FNR = 28.6%; NPV = 66.7%	NR
Heil et al. /RESPONDER (18)	Germany	2019	Prospective, multi-center	398	cPR or cCR (by imaging)	Ultrasound-guided or mammography-guided VAB (7–10G)	Mean: 7	47.7% (190/398)	Sensitivity = 82.2%; FNR = 17.8%; NPV = 81.4%	Use of 7G needles: FNR = 0%	NR

*pCR, pathologic complete response; cCR, clinical complete response; cPR, clinical partial response; CNB, core needle biopsy; VAB, vacuum-assisted biopsy; NR, not reported; TNBC, triple-negative breast cancer; NST, neoadjuvant systematic treatment; FNR, false-negative rate; NPV, negative predictive value; HR, hormonal receptor; HER2, human epidermal growth factor receptor 2; UK, United Kingdom; USA, United States of America*.

With regard to the response to NST of included patients, three of the trials only included patients with clinical complete response (cCR) by imaging and physical examination, and six of them included patients with responses not limited to cCR (plus other responses, e.g., clinical partial response, “cPR”) (Table 1).

The procedures of MIB of all of the included studies were guided by imaging methods (ultrasound, mammography, or both). Three of the studies used CNB, three of them used VAB, two used CNB and/or VAB, and one used a combination of FNA and VAB. Standard breast surgeries (lumpectomy or mastectomy) were performed following the biopsy (Table 1).

Histopathological evaluation of both MIB and surgical specimen was performed to see whether residual tumors existed. In all of the nine included trials, breast pCR was defined as the absence of cancer (both invasive and *in situ*, “ypT0”).

**Figure 2:**
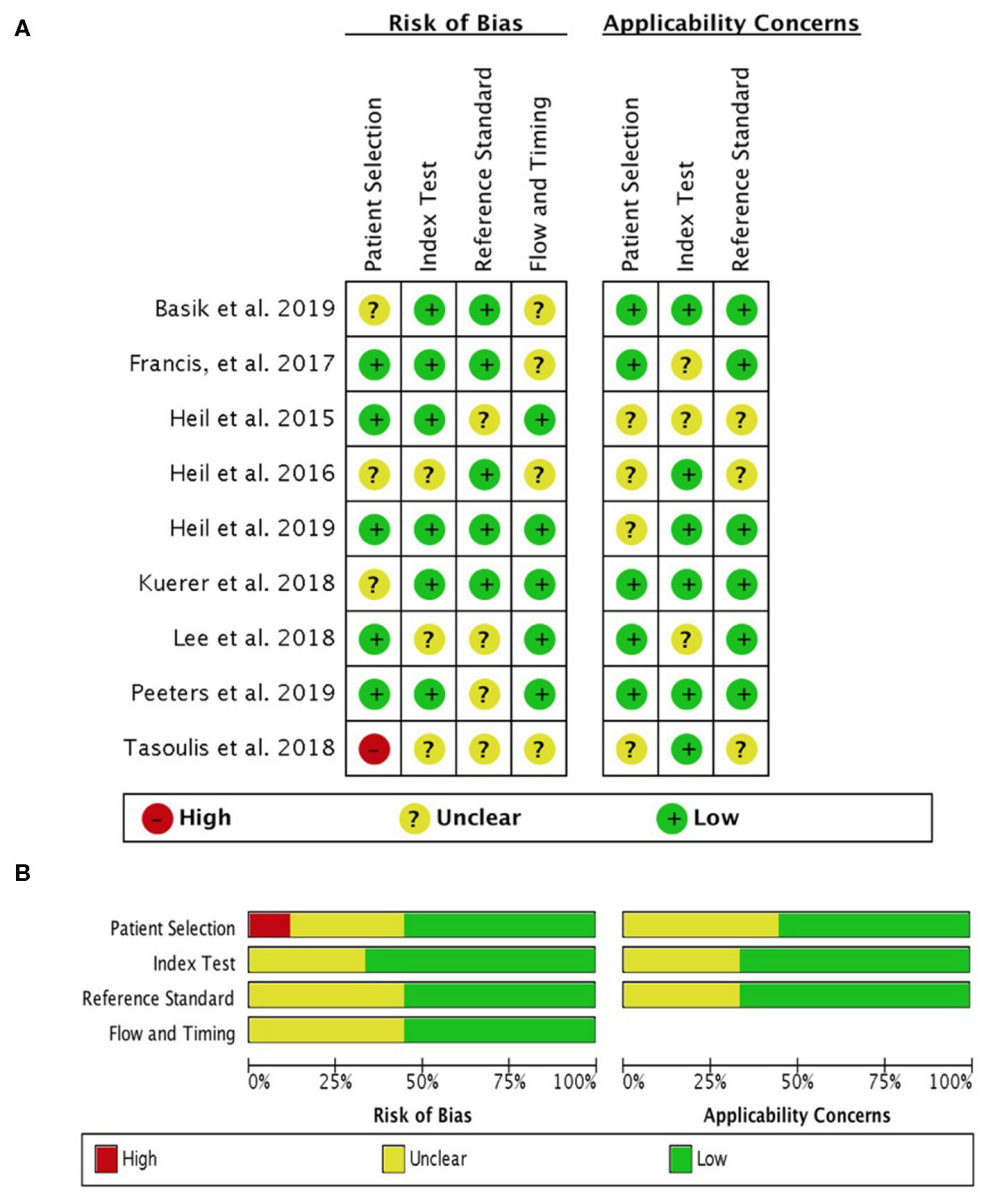
Evaluation of risk of bias by QUADAS-2 tool. **(A)** Risk of bias graph and **(B)** risk of bias summary.

The quality assessment of included studies by the QUADAS-2 tool is shown in Figure 2. One study has a high risk of bias for patient selection due to a lack of a clear description of the inclusion criteria. Risks of bias for the index test and reference test were primarily caused by a lack of reported blinding when performing pathologic examinations.

### Pooled pCR Rate According to Histopathologic Examinations of Surgical Specimens

**Figure 3:**
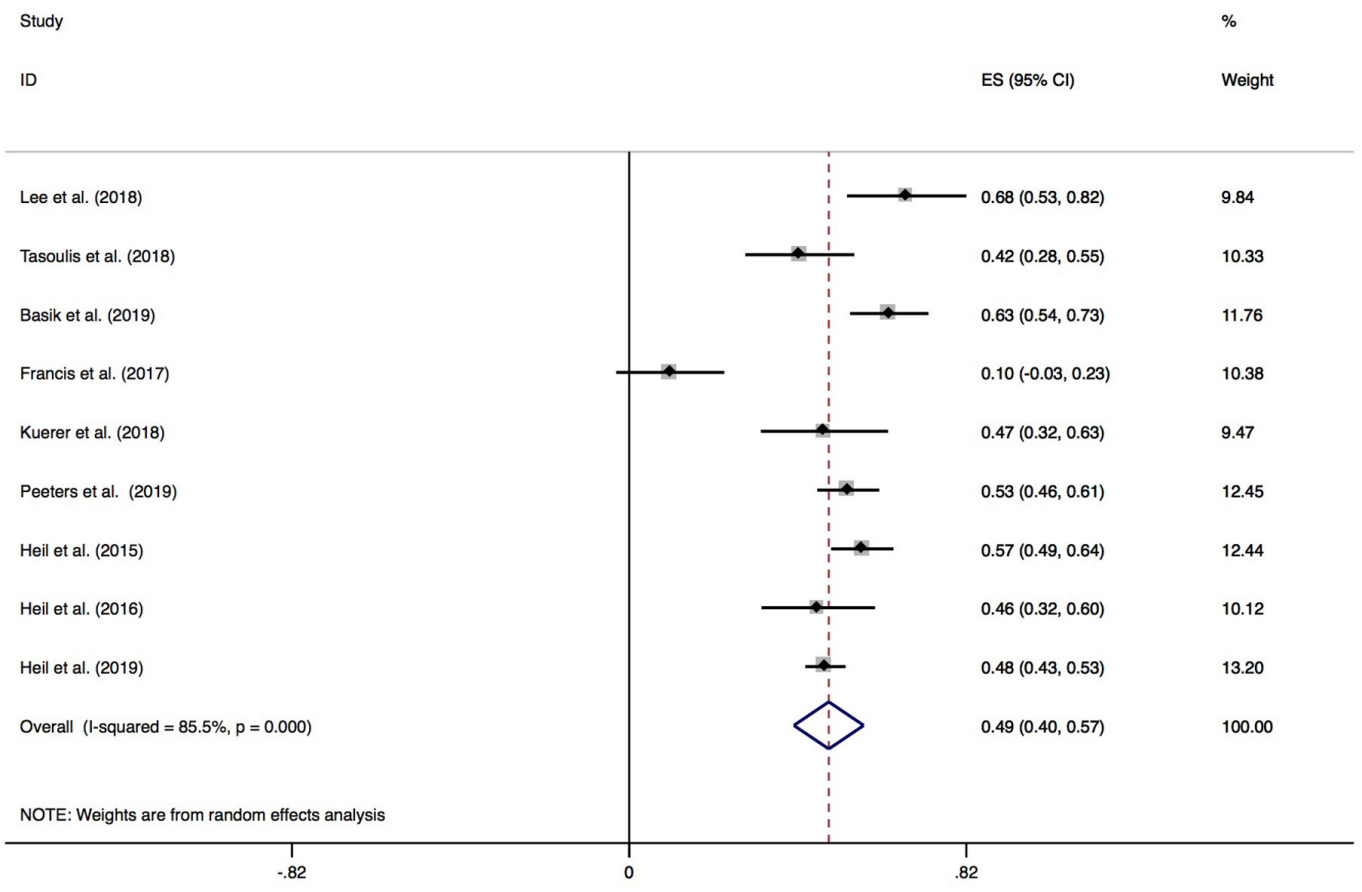
Forest plot of meta-analysis on breast pCR rate of surgical specimen.

The pCR rates confirmed by histopathologic examinations of surgical specimens of the included nine studies ranged from 10.0 to 67.5%. A random-effects model was employed due to the significant heterogeneity (*P* < 0.01, *I*^2^ = 85.5%). The pooled pCR rate was 49.0% with a 95% CI of 40.0–57.1% (Figure 3).

### Overall Diagnostic Accuracy of MIB in Predicting pCR

**Figure 4:**
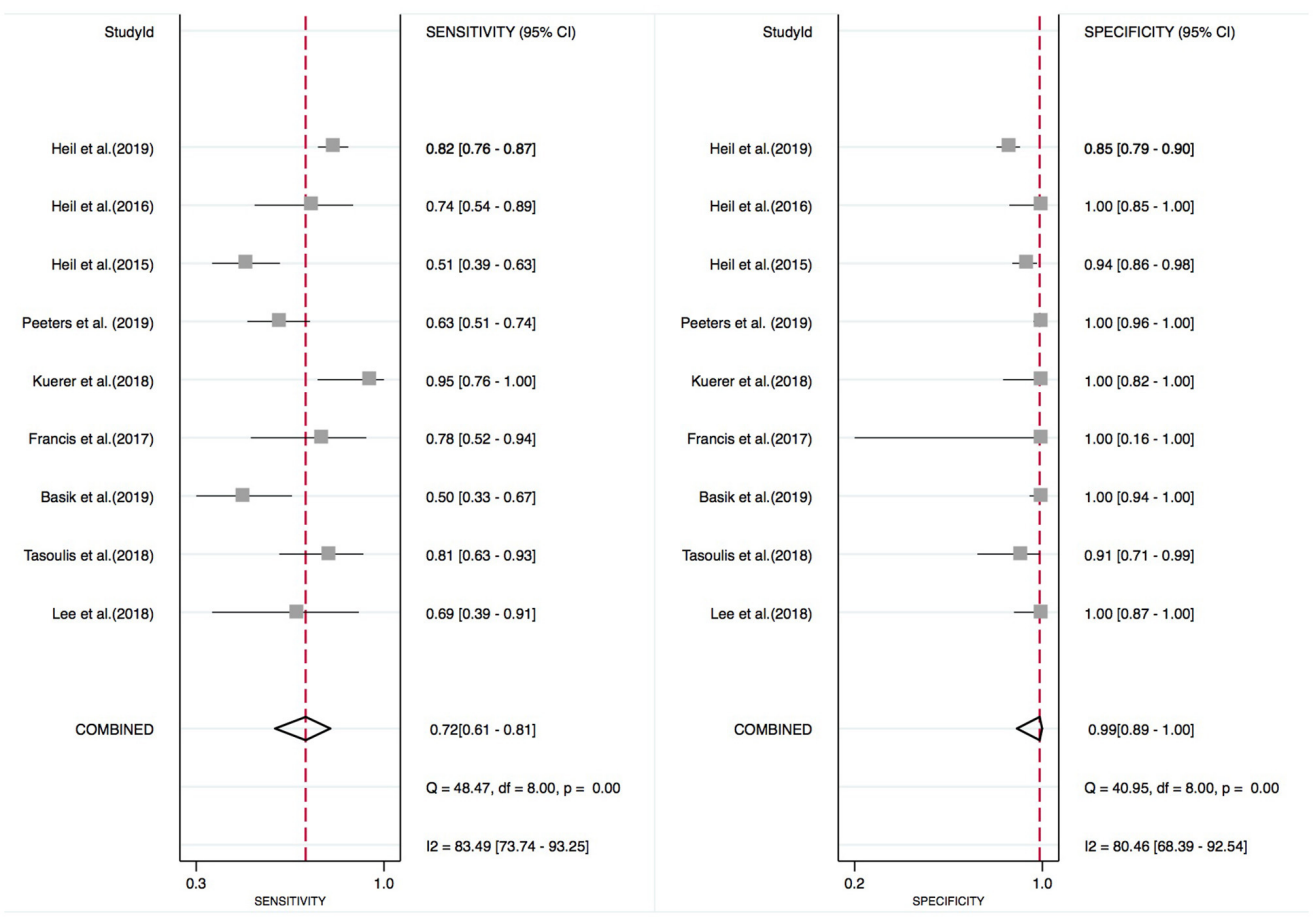
Forest plot of meta-analysis on pooled sensitivity and specificity of MIB.

Sensitivities of MIB of the included studies ranged from 50.0 to 95.0%, and specificities ranged from 85.0 to 100.0%. We selected the random effects model based on significant heterogeneity in both sensitivity and specificity (sensitivity: *P* < 0.01, *I*^2^ = 83.5%; specificity: *P* < 0.01, *I*^2^ = 80.5%). The pooled sensitivity was 0.72 with a 95% CI of 0.61–0.81. The pooled specificity was 0.99 with a 95% CI of 0.89–1.00 (Figure 4). The pooled PLR was 11.61 (5.74–23.50), the pooled NLR was 0.32 (0.23–0.44), and the pooled DOR was 38.99 (19.38–78.45).

**Figure 5:**
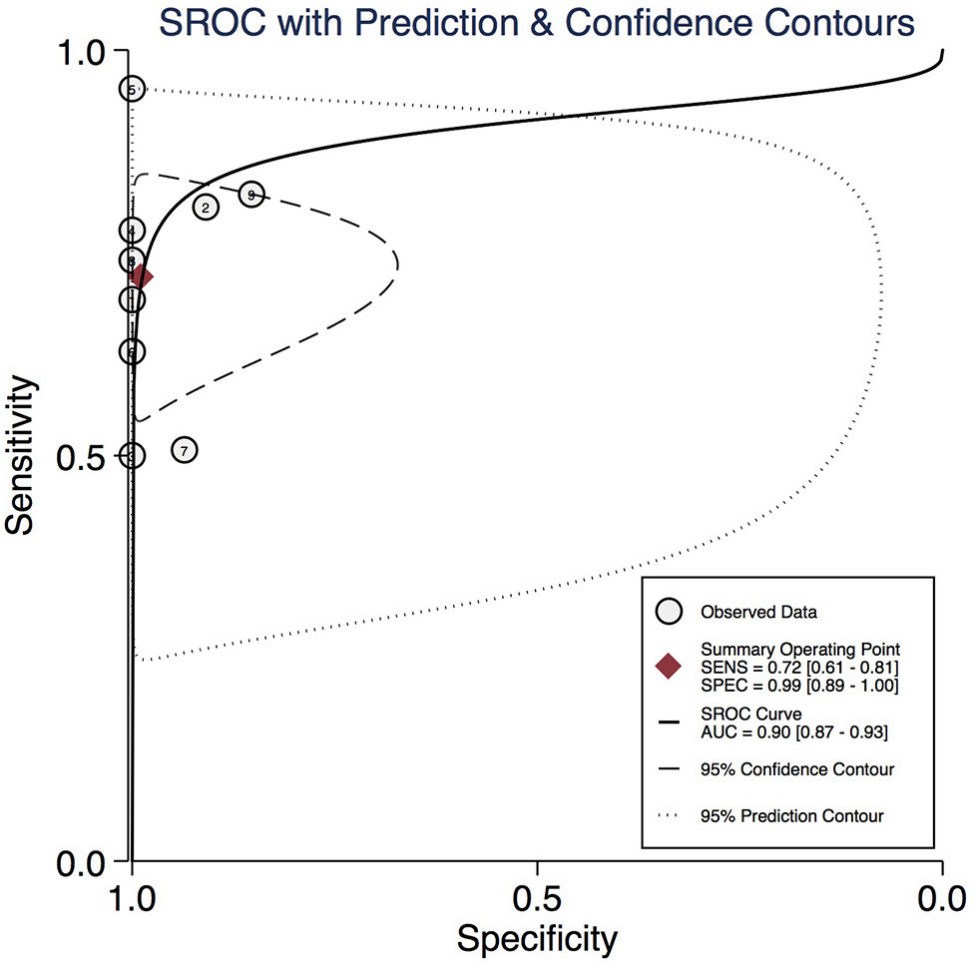
Summary receiver operator characteristic (SROC) curve of diagnostic performance of MIB.

The AUC of the SROC provides a global summary of the diagnostic performance of included studies. The AUC was 0.90 (0.87–0.93) as shown in Figure 5. An AUC score of 1.0 indicates a perfect diagnostic performance.

### Pooled Analyses According to Molecular Subtypes

Several studies described detailed information about the test accuracy of MIB in specific molecular subtypes of breast cancer (19, 22–24, 26). Table 2 shows a pooled analysis based on these data. For TNBC subtype, the pooled sensitivity was 0.47 (0.31–0.64), the pooled specificity was 0.97 (0.90–1.00), and the pooled DOR was 25.26 (6.07–105.20). For the HER2+ subtype, the pooled sensitivity was 0.59 (0.41–0.76), the pooled specificity was 0.94 (0.87–0.98), and the pooled DOR was 20.74 (3.97–108.51). For the HR+HER2− subtype, the pooled sensitivity was 0.60 (0.47–0.72), the pooled specificity was 0.94 (0.87–1.00), and the pooled DOR was 27.27 (4.84–153.76). There were no significant differences in sensitivity, specificity, or DOR among these subtypes (*P* > 0.05).

**Table 2:** Pooled analyses by molecular subtypes of breast cancer.

	**TNBC**	**HER2+**	**HR+HER2−**	**TNBC and HER2+ combined**
Number of studies	4	3	3	5
Number of patients	109	119	93	268
Pooled sensitivity (95% CI), %	47.4 (31.0–64.2)	59.4 (40.6–76.3)	59.7 (47.0–71.5)	62.6 (51.9–72.6)
Pooled specificity (95% CI), %	97.2 (90.2–99.7)	94.3 (87.1–98.1)	100.0 (86.8–100)	96.0 (92.0–98.4)
Pooled FNR (95% CI), %	52.6 (35.8–69.0)	40.6 (23.7–59.4)	40.3 (28.5–53.0)	37.4 (27.4–48.1)
Pooled PLR	8.55 (3.05–23.98)	8.30 (2.20–31.23)	11.18 (2.33–53.73)	10.30 (3.72–28.52)
Pooled NLR	0.61 (0.45–0.82)	0.48 (0.32–0.71)	0.44 (0.33–0.59)	0.31 (0.21–0.67)
Pooled DOR	25.26 (6.07–105.20)	20.74 (3.97–108.51)	27.27 (4.84–153.76)	48.73 (9.15–259.50)

*TNBC, triple-negative breast cancer; HR, hormonal receptor; HER2, human epidermal growth factor receptor 2; FNR, false-negative rare; PLR, positive likelihood ratio; NLR, negative likelihood ratio; DOR, diagnostic odds ratio; CI, confidence interval*.

### Subgroup Analysis and Meta-Regression

We also performed subgroup analysis and meta-regression according to region, study design, number of centers, response to NST, and sample size. In terms of response to NST, the studies with responses not limited to cCR had a significantly higher accuracy than those with only cCR (RDOR: 7.65; 95% CI, 1.05–55.46; *P* = 0.046). However, meta-regression analysis showed that other factors had no significant influence on the diagnostic performance of MIB (*P* > 0.05; Table 3).

**Table 3:** Subgroup analysis and meta-regression.

**Variable**	**Subgroups**	**Number of studies**	**Pooled sensitivity (95% CI),%**	**Pooled specificity (95% CI),%**	**Pooled FNR (95% CI),%**	**Pooled DOR**	**RDOR**	***P***
Region	Non-Europe	3	67.1 (54.9–77.9)	100.0 (96.6–100.0)	32.9 (22.1–45.1)	185.1 (32.2–1064.6)	0.15 (0.01–1.73)	0.106
	Europe	6	72.7 (68.3–76.9)	91.4 (88.3–93.9)	27.3 (23.1–31.7)	27.9 (15.2–51.4)		
Study design	Prospective	8	71.4 (67.1–75.4)	93.3 (90.7–95.3)	28.6 (24.6–32.9)	42.3 (28.5–96.4)	1.32 (0.06–29.00)	0.833
	Retrospective	1	80.6 (62.5–92.5)	90.9 (70.8–98.9)	19.4 (7.5–37.5)	41.7 (7.6–229.2)		
Number of centers	Single-center	5	80.0 (71.3–87.0)	97.8 (92.4–99.7)	20.0 (13.0–28.7)	69.7 (22.2–219.3)	0.38 (0.06–2.54)	0.259
	Multi-center	4	69.7 (64.9–74.2)	92.2 (89.2–94.5)	30.3 (25.8–35.1)	30.8 (12.4–76.4)		
Response to NST	Only cCR (including “near cCR”)	3	52.5 (43.2–61.7)	96.7 (93.0–98.8)	47.5 (38.3–56.8)	36.0 (7.3–177.2)	7.65 (1.05–55.46)	0.046
	cCR, cPR, and other responses	6	78.1 (73.6–82.1)	91.3 (87.8–94.1)	21.9 (17.9–26.4)	52.0 (19.5–138.8)		
Sample size	<100	6	72.6 (64.6–79.7)	98.7 (95.4–99.8)	27.4 (20.3–35.4)	75.6 (26.1–219.0)	0.35 (0.08–1.60)	0.143

*FNR, false-negative rate; DOR, diagnostic odds ratio; RDOR, relative diagnostic odds ratio; CI, confidence interval; NST, neoadjuvant systemic therapy; cCR, clinical complete response; cPR, clinical partial response*.

### Complications of MIB

Only two of the studies described complications after MIB. Basik et al. reported that 7.1% of the patients had post-procedure complications, with hematoma being the most common. However, Kuerer et al. reported a complication rate of 20.0% with bleeding as the most common, followed by hematoma [Table 1; (19, 23)].

### Publication Bias Assessment

Publication bias was not analyzed because of the limited number of included studies (29).

## Discussion

In current clinical practice, breast surgery is an important part of standard multimodal treatment for breast cancer patients after NST, and it is also the primary approach to evaluating the pathologic response to NST. Without pathologic examination of surgical specimens, a definitive pathologic diagnosis cannot be obtained. Breast-conserving surgery, followed by radiotherapy and adjuvant systemic therapy, has being confirmed safe in terms of cancer recurrence and overall survival (30). The smaller the tumor size after NST, the less tissue needs to be excised as long as there is no residual tumor in the margins (8). Thus, for patients with breast pCR after NST, complete avoidance of breast surgery is a reasonable option (11, 31). Therefore, it is extremely important to explore a non-surgical approach that can replace breast surgery in predicting pCR for breast cancer patients after NST.

Multiple studies evaluated the reliability of imaging approaches, including ultrasound, mammography, and MRI, in predicting breast pCR after NST. However, the accuracy of these techniques was far from satisfactory (12–17). Some early studies reported that using radiotherapy alone (without breast surgery) as the local treatment approach for breast cancer patients with cCR on imaging will lead to much higher rates of relapses (31–33). In recent years, there has been increasing interest in investigating the diagnostic performance of minimally invasive approaches.

To evaluate the diagnostic accuracy of image-guided MIB to identify residual cancer in the breast after NST, FNR (1-sensitivity) is a measure of paramount importance. “False negative” means that patients with the residual disease are diagnosed as “pCR,” which might lead to the incorrect omission of surgery and de-escalation of systemic therapy. On the contrary, “false positive” is of less importance because all of the residual tumors might be removed by MIB, and it is possible that the tumor was present only in the MIB specimen but not in the surgical specimen. In addition, if the MIB made a “positive” diagnosis, subsequent breast surgery is mandatory to remove potential residual disease. With regard to the maximum acceptable FNR, which will not translate into significantly worse survival outcomes, most of the included trials deduced 10% from the study design of sentinel lymph node trials (34, 35). An FNR <10% was considered acceptable although there is a lack of evidence.

This meta-analysis showed that the pooled sensitivity of image-guided MIB for the diagnosis of residual disease in the breast was 0.72, which means that the FNR was as high as 28%. An FNR of 28% in this pooled analysis is obviously far from accurate, which means that a large proportion of patients with residual disease would be diagnosed as “pCR.” Thus, breast surgery might be incorrectly omitted. Furthermore, escalation of adjuvant systemic therapy would not be administered to the patients diagnosed as “pCR.” Based on these consequences, it is reasonable to believe that missed residual tumor in the breast will eventually lead to insufficient treatment and, in the long run, lead to more relapses and worse survival outcomes. However, there is a lack of evidence about the recurrence and survival outcome of breast cancer patients with MIB-confirmed “pCR,” who forgo breast surgery accordingly. A clinical trial at MD Anderson is in the accrual phase, and the aim is to evaluate the survival consequence of eliminating breast cancer surgery in patients with VAB-confirmed “pCR” (36).

In terms of molecular subtypes of breast cancer, by combining relevant data, our pooled analysis showed that sensitivities, specificities, FNRs, and DORs had no significant differences among different subtypes. Different molecular subtypes of breast cancer showed different patterns of tumor shrinkage to NST: TNBC and HER2+ subtypes mostly exhibit concentric shrinkage, and luminal types mostly exhibit “honeycomb-like” shrinkage featured as scattered tiny foci of tumor and diffuse cell loss (37). This heterogeneous fashion of “honeycomb-like” pattern may increase the likelihood of wrong sampling of MIB, leading to low diagnostic accuracy. One of the trials included only TNBC and HER2+ subtypes and obtained a FNR of 5% (23). However, other trials reported contradictory results (19, 24). Future studies are needed to clarify the effects of molecular subtype on the accuracy of MIB.

This study also implied that trials with only response of cCR to NST had worse diagnostic accuracy than trials with responses not limited to cCR. Breast tumor evaluated as cCR by imaging methods has no clearly visible lesions. In this circumstance, MIB is always guided by clip markers placed in the tumor location prior to NST. Nevertheless, the location of clip markers is not necessarily where the tumor used to be, especially as the local tissue has changed dramatically after NST. As a result, this guidance method inevitably increases the likelihood of sampling error. For the tumor that is visible on the imaging or even palpable, it is more likely that the biopsy catches the tumor tissue. The uncertainty of clip-guided biopsy may partly explain the finding of this study, and it is helpful for further discussing the most appropriate patient group to have MIB.

It is important to notice that the procedures of MIB of the included trials varied a lot, and that is one of the main origins of heterogeneity. Many aspects of the MIB procedure can determine the diagnostic performance, including guiding imaging methods (ultrasound, mammography, and MRI), biopsy apparatus (CNB or VAB), size of needle, number of cores, and number and location of clip markers. Lee et al. reported that if more than five samples were obtained, the FNR would be around 10% (27). Heil et al. reported that using a 7G needle dramatically increases the accuracy of VAB (18). However, these included trials did not provide enough data for a pooled subgroup analysis in terms of the MIB procedure. It is of utmost importance for future studies to propose a standardized MIB procedure, which can achieve the highest predictive accuracy.

It is reported that there are other factors that are associated with pCR rates after NST for breast cancer. These factors include tumor-infiltrating lymphocytes and several specific biomarkers (38–40). Moreover, prior studies have already confirmed the predicting value of some clinical/pathological characteristics, including molecular subtypes, imaging response, and chemotherapy regimens (16, 17, 41). In the exploratory analysis of one of the included trials, Kuerer et al. reported that combining imaging appearances and MIB results can achieve an even higher predicting accuracy for pCR (23). With more and more research data accumulated, it is possible to incorporate multiple potentially relevant factors into a predictive model, which would dramatically improve the accuracy of confirming residual tumors after NST. In the construction of a predictive model with multiple parameters, artificial intelligence can play an important role (42–44). Only when a reliable non-surgical tool to rule out residual disease has been developed can omission of breast surgery be clinically feasible and safe.

The main benefit of omitting breast surgery is avoiding potential complications, better aesthetic appearance, and higher quality of life. Considering the current unaccepted accuracy of the MIB method and high possibility of missing residual disease, the benefit of surgery omission should not be achieved at the cost of oncologic safety. Furthermore, with the development of oncoplastic surgery, surgical complications, and the appearance deficits have increased significantly (45, 46). Breast surgery cannot be replaced in excellent responders of NST until the accuracy of MIB has improved to a reliable level.

There are some limitations to the present meta-analysis. First, the participants of included trials had various clinical and pathological characteristics, and these different characteristics, including age, molecular subtype, response to NST, and chemotherapy regimens, will inevitably contribute to heterogeneity. We performed meta-regressions to investigate the effects of some of these factors on pCR prediction. Second, our pooled analysis included trials with diverse MIB procedures, thereby causing bias. However, as mentioned above, there was not enough information about biopsy procedures from the included trials to make further subgroup analyses. Third, we extracted data from conference abstracts of some of the included trials, and thus, the information was not complete and may increase the difficulty of data extraction and quality assessment.

In conclusion, current image-guided MIB methods are not accurate enough in terms of predicting breast pCR after NST for breast cancer patients. The predicting accuracy is not significantly different among different molecular subtypes of breast cancer. Including patients with only cCR to NST may lead to worse prediction accuracy. It is of utmost clinical importance to standardize the MIB procedure and incorporate other factors into the evaluation in order to reduce the FNR to an acceptable level.

## Data Availability Statement

All datasets presented in this study are included in the article/supplementary files.

## Author Contributions

YLi, YZ, FM, and QS: conception and design. YLi and QS: administrative support. YLi, YZ, FM, YLin, XZ, and SS: data analysis and interpretation. All of the authors: manuscript writing and final approval of manuscript.

## Conflict of Interest

The authors declare that the research was conducted in the absence of any commercial or financial relationships that could be construed as a potential conflict of interest.
